# Vitamin D, FOXO3a, and Sirtuin1 in Hashimoto's Thyroiditis and Differentiated Thyroid Cancer

**DOI:** 10.3389/fendo.2018.00527

**Published:** 2018-09-11

**Authors:** Natascha Roehlen, Claudia Doering, Martin-Leo Hansmann, Frank Gruenwald, Christian Vorlaender, Wolf Otto Bechstein, Katharina Holzer, Klaus Badenhoop, Marissa Penna-Martinez

**Affiliations:** ^1^Division of Endocrinology, Diabetes and Metabolism, Department of Internal Medicine I, University Frankfurt, Frankfurt, Germany; ^2^Senckenberg Institute for Pathology, University Frankfurt, Frankfurt, Germany; ^3^Department of Nuclear Medicine, University Frankfurt, Frankfurt, Germany; ^4^Department of General Surgery, Buergerhospital Frankfurt, Frankfurt, Germany; ^5^Department of General Surgery, University Frankfurt, Frankfurt, Germany; ^6^Section of Endocrine Surgery, Department of Visceral, Thoracic and Vascular Surgery, Philipps University Marburg, Marburg, Germany

**Keywords:** sirtuin1, FOXO3a, vitamin D, vitamin D receptor, differentiated thyroid carcinoma, Hashimoto's thyroiditis

## Abstract

**Background:** Protective effects of vitamin D have been reported in autoimmune and malignant thyroid diseases, though little is known about the underlying mechanism. Sirtuin 1 histon deacethylase (SIRT1) links the vitamin D pathway with regulation of transcription factor FOXO3a, a key player in cell cycle regulation and apoptosis. Aim of the present study was to investigate common single nucleotide polymorphisms (SNP's) in *FOXO3a* gene in respect to thyroid diseases, as well as to evaluate the hypothesis of Sirtuin1-FOXO3a interaction being a mediator of anti-proliferative vitamin D effects.

**Methods:** The SNP's *FOXO3a* rs4946936/rs4945816/rs9400239 were genotyped in 257 patients with differentiated thyroid carcinoma (DTC), 139 patients with Hashimoto thyroiditis (HT) and 463 healthy controls (HC). Moreover, T-helper cells of HC and papillary thyroid cancer cell line BCPAP were incubated with 1,25(OH)_2_D_3_ and/or SIRT1 inhibitor Ex-527 in order to elucidate SIRT1- dependent vitamin D effects on cell proliferation and *FOXO3a* gene expression *in vitro*.

**Results:** Patients with DTC tended to carry more often allele C in *FOXO3a* rs4946936 in comparison to HC (p_corrected_ = p_c_ = 0.08). *FOXO3a* rs9400239T and rs4945816C was more frequent in HT in comparison to HC (p_c_ = 0.02 and p_c_ = 0.01, respectively). In both DTC and HT, we could not find a correlation of *FOXO3a* SNP's with vitamin D status. However, on *in vitro* level, 1,25(OH)_2_D_3_ showed an anti-proliferative effect in both T-helper cells and BCPAP, that was blocked by SIRT1 inhibition (T-helper cells: *p* = 0.0059, BCPAP: *p* = 0.04) and accompanied by elevated *FOXO3a* gene expression in T-helper cells (*p* = 0.05).

**Conclusions:** *FOXO3a* rs9400239T and rs4945816C may constitute risk factors for HT, independent of the vitamin D status.This indicates the implication of FOXO3a in pathogenesis of autoimmune thyroid diseases. The dependency of anti-proliferative vitamin D effects on SIRT1 activity further suggests a key role of vitamin D-SIRT1-FOXO3a axis for protective vitamin D effects.

## Introduction

Hashimoto's thyroiditis (HT) and differentiated thyroid cancer (DTC) are the most common autoimmune and malignant thyroid diseases. Although they differ by pathophysiology, both diseases manifest on the background of genetic susceptibility and either triggering or causal environmental factors [1, 2]. These etiological characteristics become apparent in the frequent coincidence of HT with DTC, as well as in the familial clustering of the disease [3, 4].

Vitamin D (VD) deficiency represents a joint risk factor for HT [5] and DTC [6] and affects ~50% of the global population [7]. Thus, low levels of vitamin D have been found in HT [5] and DTC [8]. Notably, an inverse correlation of VD levels was found with the malignancy rate of thyroid nodules [6] as well as with thyroid antibody titers [1, 9] suggesting a causal link.

Whereas 25(OH)D_3_ is the stable inactive metabolite and used to assess the VD status in humans, 1,25(OH)_2_D_3_ is the final active form of VD, interacting with the VD receptor (VDR). By targeting various transcription factors and cell cycle proteins, the VDR mediates anti-proliferative, pro-differentiating and immune-modulating effects [10]. Thus, VD was reported to inhibit cell growth of thyroid cancer (TC) cells [11]. Moreover, VD has been shown to regulate type 1 and 17 T helper cells [12, 13], that are involved in the pathogenesis of HT [14, 15]. Appropriately, VD treatment in patients with HT has been shown to decrease levels of thyroid antibodies (Ab's) [16]. Various associations have been reported for single nucleotide polymorphisms (SNP's) of the VDR or VD metabolizing enzymes with both DTC [17, 18] and HT [19, 20]. Therefore VD may further impact on autoimmune and malignant thyroid diseases through genetic susceptibility. However, until now the exact molecular mechanisms of VD's preventive potential in HT and DTC are still unclear.

Sirtuins are histone deacetylases, which are involved in various metabolic pathways, including aging and stress response [21]. As NAD dependent metabolic sensors, sirtuins adjust posttranslational modification of cellular regulator proteins on energy status of the cell [22]. Sirtuin 1 (SIRT1) represents a subform located in the nucleus that has lately not only been shown to be regulated by the VDR, but also been interpreted as a mediator of anti-proliferative VD signals by deacetylation of forkhead box protein O3a (FOXO3a) [23]. SNP's of the FOXO3a related transcription factor forkhead box protein E1 (FOXE1) have only recently been reported as important risk factors for DTC [24]. Interestingly, posttranslational modification of FOXO3a by SIRT1 antagonizes the phosphatidylinositol-4,5-bisphosphate 3-pathway [25]. This signal cascade is activated in T-helper cells (Thc) upon T cell receptor activation [26] and has been found to be oncogenic in thyroid carcinoma [27]. This suggests a potential key role of VD-SIRT1-FOXO3a signaling in immune regulation and DTC.

To the best of our knowledge, the VD-SIRT1-FOXO3a interaction has never been investigated in relation to autoimmune and malignant thyroid diseases. In this study, we aimed to examine common *FOXO3a* SNP's for association with autoimmune or malignant thyroid diseases. Moreover we questioned the SIRT1-dependency of anti-proliferative VD effects on immune cells and malignant thyroid cells as an indication of VD-SIRT1-FOXO3a interaction constituting a molecular pathway of protective VD effects.

## Materials and methods

### Subjects

In total 257 patients (172 females and 85 males, median age: 55 years) with pathologically confirmed diagnosis of DTC (208 of papillary differentiation and 49 of follicular differentiation) as well as 139 patients with HT (116 females and 23 males, median age: 40 years) were recruited from the Department of Medicine 1 and the Department of Nuclear Medicine at the University Hospital Frankfurt am Main, Germany as well as from the Department of Surgery at the Bürgerhospital Frankfurt am Main, Germany. Furthermore 463 healthy controls (HC, 228 females and 235 males, median age: 37 years) were volunteer blood donors.

The study protocol was approved by the Ethics Committee of the University Hospital Frankfurt am Main. Informed and written consent was obtained from all participants.

## Methods

### Measurement of vitamin D metabolites and thyroid antibodies

The 25(OH)D_3_ and 1,25(OH)_2_D_3_ plasma levels were measured by radioimmunoassay (DiaSorin, Stillwater, Minnesota, USA and IDS, Frankfurt am Main, Germany) in blood samples of DTC, HT, and HC. The expected normal range (95% reference interval) in healthy adults using this method is 9.0–37.6 ng/ml for 25(OH)D_3_ and 18.06–70.56 pg/ml for 1,25 (OH)_2_D_3_ level with a reported coefficient of variance of < 20% and < 11%, respectively. An enzyme-linked immunosorbent assay (Phadia, Freiburg, Germany) was used to determine serum levels of thyroglobulin (Tg)- and thyroperoxidase (TPO)- Ab's in patients with HT (*n* = 91).

### Single nucleotide polymorphism analysis

The *FOXO3a* gene is located on chromosome 6 at the gene locus q21 (6q21). Three single nucleotide polymorphisms (SNP's) within *FOXO3a* gene were studied in DTC, HT and HC. The SNPs rs4946936 and rs4945816 are located at the 3′ untranslated (UTR) region of *FOXO3a*. The third SNP rs9400239 is located at the 5′ end of *FOXO3a*.

Genomic DNA from all blood samples was isolated by Miller and Dakes salting out procedure and subsequently amplified by polymerase chain reaction. The SNP's within the *FOXO3a* gene rs4946936 and rs4945816 (both specifically designed by Applied Biosystems) and rs9400239 (Assay-ID: C__11904122_10, Applied Biosystem) were analyzed using Taqman assays in an ABI 7300 system with conditions, recommended by the manufacturer. Random samples of the *FOXO3a* rs4946936 and *FOXO3a 9400239* were analyzed twice in order to confirm the accuracy of the applied method. The results revealed a concordance rate of 100%.

### Isolation of T-helper cells

Ten HC, consisting of 5 men and 5 women (median age: 26 years) were recruited for analysis of physiological VD–SIRT1-FOXO3a pathway in immune cells. Therefore, Thc were isolated from peripheral blood mononuclear cells of the subjects by using the CD4 T cell Isolation Kit II (Miltenyi Biotec, Bergisch Gladbach, Germany). During cell cultivation in basic cell medium, all cells were supplemented with the T cell mitogen phytohemagglutinin (PHA, Sigma-Aldrich) in concentrations of 50 μg/ml.

### Cell line preparation

Human papillary thyroid cancer cell line BCPAP (RRID:CVCL_0153) was obtained from Leibniz Institute DSMZ, German Collection of Microorganisms and Cell Cultures (Braunschweig, Germany). This cell line was authenticated by DSMZ using multiplex PCR of minisatellite markers. The cells were processed immediately upon receipt and cells of passage four were used for five repetitions of the following cell culture approaches.

### Cell cultivation

All cells were cultivated in RPMI-1640, that was supplemented with 10% FCS, 1% penicillin-streptomycin and 25 mM Hepes (basic cell medium) and were seeded out in volumes of 5 × 10^5^ cells/ml in 6 well plates, in case of BCPAP cells (Sigma-Aldrich, munich, Germany, growth area: 9.5 cm^2^) or 24 well plates in case of Thc (Sigma-Aldrich, growth area: 1.9 cm^2^). Furthermore, either 10 nM 1,25(OH)_2_D_3_ or 2.4 μM of the SIRT1 inhibitor Ex-527 [that have been reported to abolish SIRT1 activity by virtually 100%, respectively [28]] or the combination of 1,25(OH)_2_D_3_ and Ex-527 were added.

The cells were incubated at 37°C. Forty-eight and ninety-six hours after isolation, cells were detached from the plates, requiring trypsinization in case of thyroid cells. Finally all cells were counted using hemocytometer.

### Gene expression analysis

A total of 18 ng RNA was extracted from BCPAP cells and Thc for reverse transcription into cDNA (Affinity Script QPCR, Agilent Technologies, Waldbronn, Germany). Quality control of RNA-integrity was performed previous to reverse transcription, using 2100 Bioanalyzer (Agilent Technologies). Subsequently, gene expression of *FOXO3a* (Hs00921424_m1) and the endogenous control 18s (Hs99999901_s1) were measured by Taqman assay (Applied Biosystems). Gene expression were compared as relative levels of cycle threshold (CT) normalized to the endogenous controls, calculated by 2^−[CTtarget(t)−CT18s(t)]^ × 10^6^.

### Statistical analysis

The statistical analyses were performed using BiAS statistic software package 10.0 (Epsilon, Weinheim, Germany). Differences in genotype and allele distributions between all groups were evaluated by chi-square test. The odds ratio (OR) and its 95% confidence interval (CI) were estimated by unconditional logistic regression. CT-values, levels of thyroid Ab's, and cell proliferation were analyzed by non-parametric tests (Willcoxon-Mann-Whitney-U-test, Kruskall-Wallis-test and Wilcoxon matched pairs test).

In case of multiple comparisons, *p* values were corrected by multiplying them with the number of comparisons tested (Bonferroni correction). *P*-values (p or p_c_, respectively) < 0.05 were considered significant, *p*-values (p or p_c_, respectively) < 0.10 were considered as a trend.

## Results

### *FOXO3a* single nucleotide polymorphisms in patients with differentiated thyroid carcinoma

DTC and HC were genotyped for the SNP's *FOXO3a* rs4946936, rs4945816, and rs9400239. The genotypes of all groups were in Hardy Weinberg equilibrium (*p* > 0.05).

The genotype analyses of all three SNP's did not show any differences between DTC and HC (Supplemental Table 1). However allele analysis of *FOXO3a* rs4946936 revealed a tendency of a higher frequency of allele “C” in DTC in comparison to HC (“C”: DTC vs. HC = 72.4% vs. 66.7%, OR: 1.28[CI 0.99–1.66], p_c_ = 0.08, Supplemental Table 1). Allele and genotype distributions did not show association with markers of malignancy in DTC (tumor grade, TNM classification). Moreover, no correlation was found between genotypes of all investigated SNP's and VD status (DTC: Median level: 25(OH)D_3_ = 17.0 ng/ml and 1,25(OH)_2_D_3_ = 36.0 pg/ml; HC: 25(OH)D_3_ = 19.95 ng/ml and 1,25(OH)_2_D_3_ = 52.7 pg/ml, consistent within all genotypes, data not shown).

### *FOXO3a* single nucleotide polymorphisms in patients with hashimoto thyroiditis

*FOXO3a* rs4946936, rs4945816, and rs9400239 were analyzed in 139 HT. Genotype analysis showed elevated frequency of “TT” in rs9400239 and “CC” in rs4945816 in patients with HT (*FOXO3a* rs9400239: “TT”: HT vs. HC = 16.5 vs. 9.3%, p_c_ = 0.06; *FOXO3a* rs4945816: “CC”: HT vs. HC = 15.1 vs. 8.8%, p_c_ = 0.06, Table 1). Moreover, HT carried allele T in rs9400239 and allele C in rs4945816 significantly more often than HC (*FOXO3a* rs9400239: “T”: HT vs. HC = 39.9 vs. 31.4%, OR: 1.45 [CI 1.10–1.91], p_c_ = 0.02; *FOXO3a* rs4945816: “C”: HT vs. HC = 39.6 vs. 30.5%, OR: 1.5 [CI 1.12–2.00], p_c_ = 0.01). These associations were especially strong in males (*FOXO3a* rs9400239: “T”: HT♂ vs. HC♂ = 52.2 vs. 30.9%, OR: 2.05 [CI 1.12–3.78], p_c_ = 0.04, *FOXO3a* rs4945816: “C”: HT♂ vs. HC♂ = 47.8 vs. 29.9%, OR: 2.15 [CI 1.15–4.04], p_c_ = 0.04, Table 1). We also analyzed the Tg- and TPO Ab's levels in relation to *FOXO3a* rs9400239 and rs4945816 genotypes. However we could not find significant differences (data not shown).

**Table 1:** Distribution of *FOXO3a* single nucleotide polymorphisms in Hashimoto thyroiditis and healthy controls.

**Genotype/** ***n*** **(frequency)**						**Allele/** ***n*** **(frequency)**						
**Group**	***n***	**CC**	**CT**	**TT**	***p***	**pc**	**C**	**T**	**OR [95% CI] C**	**OR [95% CI] T**	***p***	**pc**
**FOXO3a rs4946936**												
HC	362	156 (43.1%)	171 (47.2%)	35 (9.7%)			483 (66.7%)	241 (33.3%)				
HT	139	50 (36.0%)	70 (50.4%)	19 (13.7%)	0.23	0.69	170 (61.2%)	108 (38.8%)	0.79 [0.59–1.05]	1.27 [0.96–1.7]	0.11	0.22
HC♀	208	89 (42.8%)	99 (47.6%)	20 (9.6%)			277 (66.6%)	139 (33.4%)				
HT♀	116	43 (37.1%)	59 (50.9%)	14 (12.1%)	0.55	1.65	145 (62.5%)	87 (37.5%)	0.84 [0.60–1.17]	1.20 [0.86–1.67]	0.34	0.68
HC♂	154	67 (43.5%)	72 (46.8%)	15 (9.7%)			206 (66.9%)	102 (33.1%)				
HT♂	23	7 (30.4%)	11 (47.8%)	5 (21.7%)	0.19	0.57	21 (45.7%)	25 (54.3%)	0.59 [0.31–1.10]	1.74 [0.91–3.18]	0.13	0.26
**FOXO3a rs4945816**												
HC	353	31 (8.8%)	153 (43.3%)	169 (47.9%)			215 (30.5%)	491 (69.5%)				
HT	139	21 (15.1%)	68 (48.9%)	50 (36.0%)	**0.02**	**0.06**	110 (39.6%)	168 (60.4%)	1.50 [1.12–2.00]	0.67 [0.50–0.89]	**0.007**	**0.01**
HC♀	204	17 (8.3%)	92 (45.1%)	95 (46.6%)			126 (30.9%)	282 (69.1%)				
HT♀	116	14 (12.1%)	60 (51.7%)	42 (36.2%)	0.17	0.51	88 (37.9%)	144 (62.1%)	1.37 [0.98–1.92]	0.73 [0.52–1.03]	0.08	0.16
HC♂	146	14 (9.4%)	61 (40.9%)	71 (49.7%)			89 (29.9%)	209 (70.1%)				
HT♂	23	7 (30.4%)	8 (34.8%)	8 (34.8%)	**0.01**	**0.04**	22 (47.8%)	24 (52.2%)	2.15 [1.15–4.04]	0.46 [0.25–0.87]	**0.02**	**0.04**
**FOXO3a rs9400239**												
HC	463	215 (46.4%)	205 (44.3%)	43 (9.3%)			635 (68.6%)	291 (31.4%)				
HT	139	51 (36.7%)	65 (46.8%)	23 (16.5%)	**0.02**	**0.06**	167 (60.1%)	111 (39.9%)	0.69 [0.52–0.91]	1.45 [1.10–1.91]	**0.01**	**0.02**
HC♀	228	104 (45.6%)	102 (44.7%)	22 (9.7%)			310 (68.9%)	146 (32.9%)				
HT♀	116	43 (37.1%)	57 (49.1%)	16 (13.8%)	0.24	0.72	143 (61.6%)	89 (38.4%)	0.76 [0.54–1.05]	1.32 [0.95–1.84]	0.11	0.22
HC♂	235	111 (47.2%)	103 (43.8%)	21 (8.9%)			325 (69.1%)	145 (30.9%)				
HT♂	23	7 (30.4%)	8 (34.8%)	8 (34.8%)	**0.006**	**0.01**	22 (47.8%)	24 (52.2%)	0.49 [0.26–0.90]	2.05 [1.12–3.78]	**0.02**	**0.04**

*The single nucleotide polymorphisms FOXO3a rs4946936, rs4945816 and rs9400239 were genotyped in patients with HT (n = 139) and HC (n = 463). HT showed higher frequency of minor allele “T” in rs9400239 and “C” in rs4945816 in comparison to HC. The differences were especially strong in male patients leading to an elevated risk of 2.05 in male carriers of rs9400239T and of 2.15 in male carriers of rs4945816C. Significant asociations are displayed in bold*.

The genotypes and alleles of *FOXO3a* rs4946936 did not differ between HT and HC.

No correlation was found between genotypes of all investigated SNP's and VD status (HT: Median level: 25(OH)D_3_ = 18.35 ng/ml and 1,25(OH)_2_D_3_ = 51.0 pg/ml, consistent within all genotypes, data not shown).

### SIRT1-dependent VD effects on cell proliferation in T-helper cells

Since FOXO3a was reported as a target of the VD cascade by mediation of SIRT1, the effects of VD on cell proliferation were analyzed in relation to SIRT1 activity. Hereby, Thc of HC served as a model of physiologic VD-SIRT1-FOXO3a interaction.

**Figure 1:**
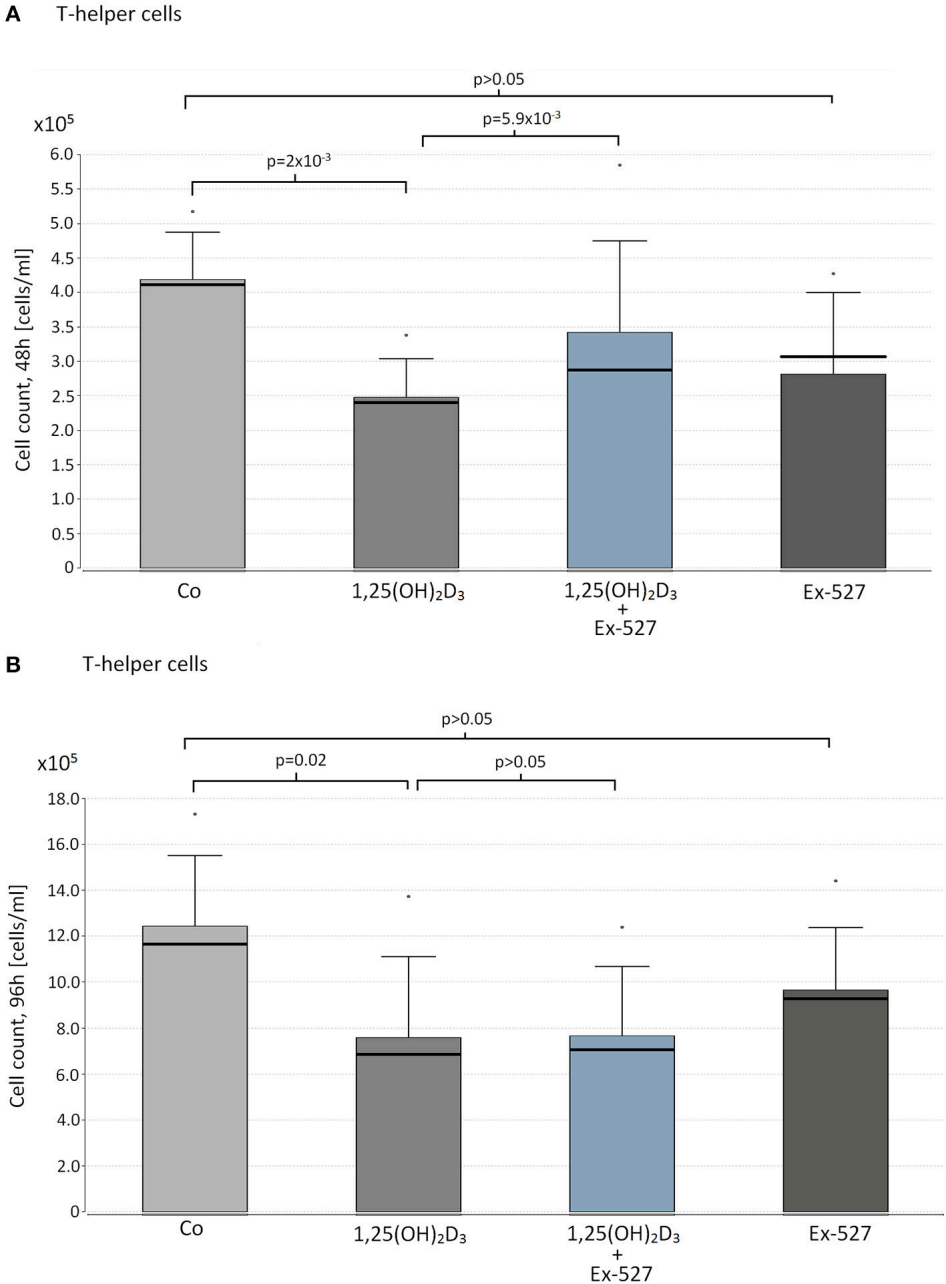
Effect of vitamin D supplementation and SIRT1 inhibition on proliferation on T-helper cells. Thc of 10 HC were supplemented with the following reagents in cell counts of 1 x 10^5^ cells/ml: 10 nM 1,25(OH)_2_D_3_ or 2.4 μM Ex-527, or 10 nM 1,25(OH)_2_D_3_+2.4 μM Ex-527. After 48 h **(A)** and 96 h **(B)** of cell cultivation, cell proliferation was measured by hemocytometer. Figure shows the mean value (bar level), the median (bold line), the standard deviation and maximum level of the measured cell counts in the corresponding groups of cell approaches.

We observed a significant cell suppressive effect of 1,25(OH)_2_D_3_ on Thc after 48 and 96 h (Median cell count(48 h): 1,25(OH)_2_D_3_ vs. Co = 2.48 × 10^5^ vs. 4.16 × 10^5^ cells/ml, *p* = 0.002, Figure 1A, 96 h: 1,25(OH)_2_D_3_ vs. Co = 6.86 × 10^5^ vs. 1.15 × 10^6^, *p* = 0.02 Figure 1B). Interestingly the cell suppressive effect of VD was blocked in cells that were additionally supplemented with the SIRT1 inhibitor Ex-527 after 48 h (Ex-527+1,25(OH)_2_D_3_ vs. 1,25(OH)_2_D_3_ = 2.81 × 10^5^ vs. 2.48 × 10^5^ cells/ml, *p* = 0.0059, Figure 1A).

Supplementation with Ex-527 alone did not affect cell count after 48 and 96 h of cell cultivation (Figures 1A,B).

### SIRT1-dependent VD effects on cell proliferation in cell line BCPAP

**Figure 2:**
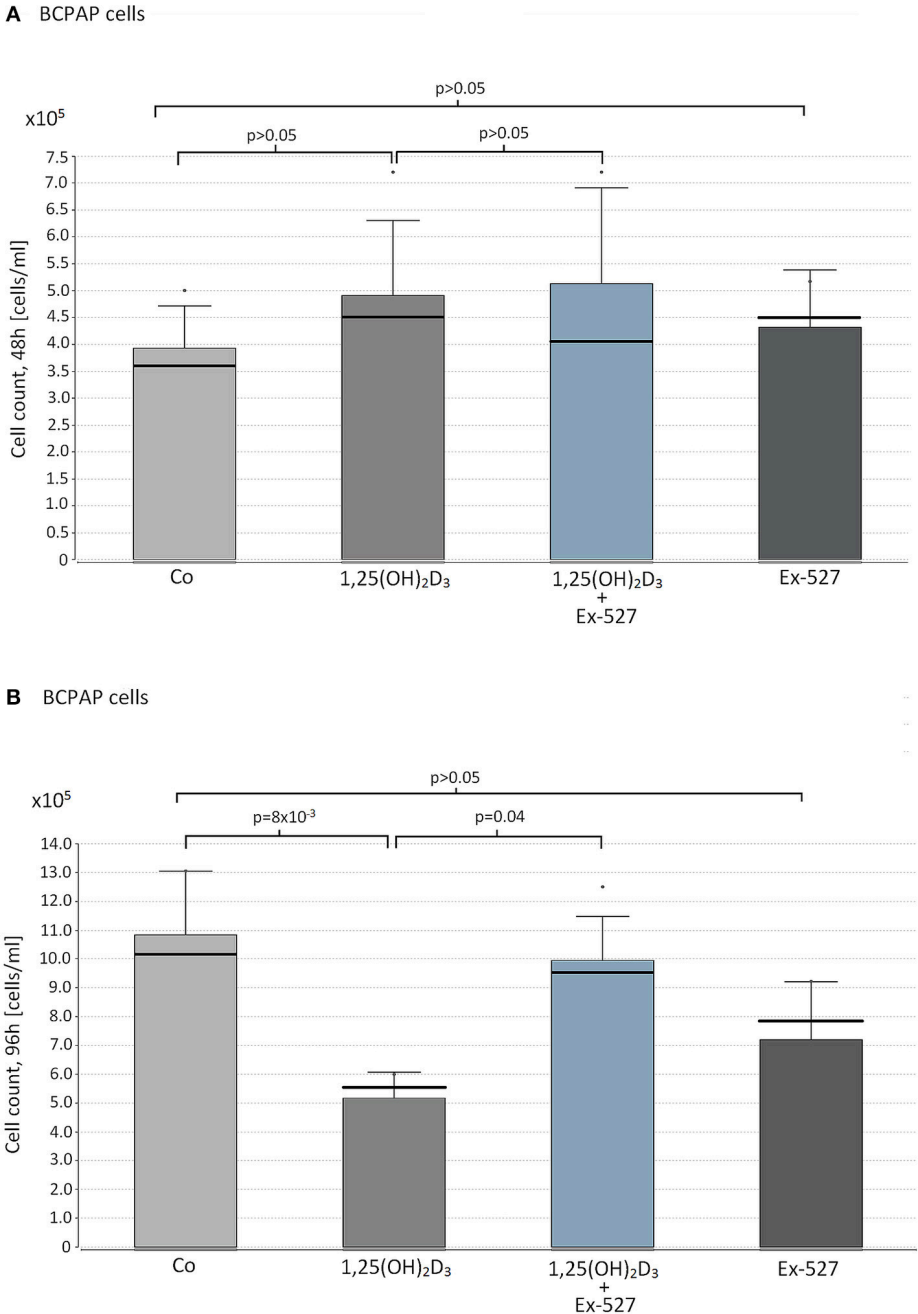
Effect of vitamin D supplementation and SIRT1 inhibition on proliferation on papillary thyroid cancer cells. Papillary thyroid cancer cells of cell line BCPAP were supplemented with the following reagents in cell counts of 1 × 10^5^ cells/ml: 10 nM 1,25(OH)_2_D_3_ or 2.4 μM Ex-527, or 10 nM 1,25(OH)_2_D_3_+2.4 μM Ex-527. After 48 h **(A)** and 96 h **(B)** of cell cultivation, cell proliferation was measured by hemocytometer. Figure shows the mean value (bar level), the median (bold line), the standard deviation and maximum level of the measured cell counts in the corresponding groups of cell approaches.

Based on the insights of physiological VD/SIRT1/FOXO3a interplay in immune cells, a similar experimental setup was carried out in papillary thyroid cancer cells. None of the reagents affected BCPAP cell proliferation within 48 h of cultivation (1,25(OH)_2_D_3_ vs. Co; 1,25(OH)_2_D_3_+Ex-527 vs. 1,25(OH)_2_D_3_, Ex-527 vs. Co, p_each_>0.05, Figure 2A). However, as seen for Thc, we observed a significant inhibitory effect of 1,25(OH)_2_D_3_ on cell proliferation after 96 h (1,25(OH)_2_D_3_ vs. Co = 5.6 × 10^5^ vs. 1.2 × 10^6^ cells/ml, *p* = 8 × 10^−3^). Moreover, once again addition of SIRT1 inhibitor Ex-527 to 1,25(OH)_2_D_3_ treated cells reduced the anti-proliferative effect of 1,25(OH)_2_D_3_ (1,25(OH)_2_D_3_+Ex-527 vs. 1,25(OH)_2_D_3_ = 9.5 × 10^5^ vs. 5.6 × 10^5^ cells/ml, *p* = 0.04, Figure 2B). SIRT1 inhibition by Ex-527 alone did not affect cell proliferation of BCPAP after 48 and 96 h of cell cultivation (Figures 2A,B).

### SIRT1-dependent VD effects on *FOXO3a* gene expression in T- helper cells and cell line BCPAP

Thc and BCPAP were further analyzed for SIRT1 dependent VD effects on *FOXO3a* gene expression level. Interestingly, VD supplemented Thc showed significant increase in *FOXO3a* gene expression after 48h (1,25(OH)_2_D_3_ vs. Co: 20.52 vs. 13.28 2^−(CT FOXO3a−CT 18s)^ × 10^6^, *p* = 0.05, Table 2). Remarkably, Thc cultivated with VD+Ex-527 did not differ in *FOXO3a* gene expression levels from Thc supplemented with 1,25(OH)_2_D_3_ alone (1,25(OH)_2_D_3_ vs. 1,25(OH)_2_D_3_+Ex-527: 20.52 vs. 21.66 2^−(CTFOXO3a−CT 18s)^x10^6^, *p* > 0.05, Table 2). After 96 h of cultivation no effects were seen on *FOXO3a* gene expression for neither VD supplementation, nor the combination of VD and Ex-527 (data not shown).

**Table 2:** Effect of vitamin D stimulation and SIRT1 inhibition on *FOXO3a* gene expression in T-helper cells and BCPAP cells.

**48 h**	**T-helper cells**		**BCPAP cells**	
**Stimulation**	***FOXO3a* gene expression Median [2^−(CTFOXO3a−CT 18s)^x 10^6^]**	***p***	***FOXO3a* gene expression Median [2^−(*CTFOXO*3*a*−*CT* 18*s*)^x 10^6^]**	***p***
Co	13.28		98.47	
1,25(OH)_2_D_3_	20.52	**0.05**	99.84	0.81
1,25(OH)_2_D_3_	20.52		99.84	
Ex-527+1,25(OH)_2_D_3_	21.66	0.73	76.19	0.13

*PHA-stimulated T-helper cells and B-CPAP cells were cultivated in 5 × 10^5^ cell approaches with 1,25(OH)_2_D_3_ or SIRT1 inhibitor Ex-527 or 1,25(OH)_2_D_3_ + Ex-527. After 48h of cultivation FOXO3a gene expression was measured by real time PCR and compared to cell approaches without any additions (Co). 1,25(OH)_2_D_3_ increased FOXO3a gene expression significantly after 48 h of supplementation. Significant asociations are displayed in bold*.

In contrast to Thc, VD supplementation did not affect *FOXO3a* gene expression in BCPAP. Also the combination of VD and SIRT1 inhibitor did not alter *FOXO3a* gene expression in papillary TC cells (Table 2).

## Discussion

Protective effects of vitamin D have been reported in several autoimmune [29] and malignant diseases [30], though little is known about the underlying molecular mechanisms.

In thyroid diseases, out of the VD pathway SNP's in the VDR are the best-studied genetic associations with HT and DTC. Four polymorphisms detected by restriction enzymes FokI, BsmI, ApaI, and TaqI have been investigated extensively in relation to thyroid diseases. Changes of the predicted VDR protein sequence at the FokI and ApaI sites have been associated with DTC [18] whereas FokI, BsmI, and TaqI have been related to autoimmune thyroid diseases [31–33].

New aspects of VD's mode of action affect SIRT1, a histone deacetylase implicated in cancer formation [34, 35] and autoimmune mechanisms [36]. Enzymatic activation of SIRT1 by the VDR mediates deacetylation and hereby reactivation of the transcription factor FOXO3a [23]. Interestingly, this forkheadbox protein is also found to be inhibited in DTC [37] and Thc upon stimulation [26]. This concurrence suggests that FOXO3a could be a key mediator of VD's anti-proliferative and immune regulatory effects, also affecting the pathogenesis of DTC and HT. Since genetic variations of other members of the forkhead box protein family have already been identified as important risk factors for thyroid diseases [24], we questioned the association of *FOXO3a* SNP's with DTC and HT. Thus, *FOXO3a* rs4946936T has already been reported to be associated with vitiligo, an autoimmune disease that often occurs together with HT [38]. In fact, *FOXO3a* rs9400239T and rs4945816C were associated with HT in our study group. To our knowledge, this is the first report of *FOXO3a* SNP's showing associations with autoimmune thyroid disease. Due to the limited number of patients, these observations need to be confirmed in a larger cohort. Since, rs9400239T or rs4945816C did not correlate with the Tg- or TPO-Ab level in HT, the underlying pathomechanism may not directly impact on antibody related immunological mechanisms.

Consistent with recent analyses of *FOXO3a* SNP's in cancers of other origin [39], we only found tendencies toward a higher frequency of *FOXO3a* rs4946936C in DTC. Future studies with more probands should elucidate the relevance of the observed distributions.

Despite its function as a target of the VD cascade, the association of *FOXO3a* SNP's with HT did not correlate with the VD status of the patients. In line with this observation, the *FOXO3a* gene itself does not harbor any VDRE's and FOXO3a transcriptional control is thought to occur via a VDRE in a neighboring gene [40].

A molecular explanation for the functional relevance of those non-coding SNP's could be an interaction with micro RNA's [41] affecting *FOXO3a* mRNA expression levels [42]. Consistent with this observation, *FOXO3a* gene expression has been reported to be dysregulated in DTC [37].

Next we turned to the *in vitro* situation and questioned the dependency of protective VD effects on SIRT1-FOXO3a axis. In fact, our investigations indicate anti-proliferative effects of 1,25(OH)_2_D_3_ to depend on SIRT1 activity in Thc and cancer cells. Thus, the presence of the SIRT1 inhibitor Ex-527 in concentrations, known to abolish SIRT1 activity by virtually 100% [28], reduced the cell suppressive effect of 1,25(OH)_2_D_3_ on BCPAP and Thc. Our results therefore indicate SIRT1 to be a crucial component of VD's anti-proliferative signals in thyroid cancer cells and immune cells. Appropriately, those cell cycle regulative and pro-apoptotic genes that have been attributed as molecular mediators of VD's protective effects, such as Bim or GADD45 [43, 44], are also induced by SIRT1 activated FOXO3a [45, 46]. In line with our investigations in DTC and immune cells, Sabir et al. only recently reported attenuation of VD signaling by Ex-527 in two different human non-thyroid cell lines [47]. Furthermore An et al. linked VD signaling with enhanced cell cycle control by FOXO3a [23].

Our analyses further revealed a SIRT1 independent interaction of FOXO3a and the VD pathway in Thc. Thus, we observed a stimulative effect of 1,25(OH)_2_D_3_ on *FOXO3a* transcription in Thc, that wasn't affectable by SIRT1 inhibition. In contrast, this effect was not seen in papillary thyroid cancer cells of cell line BCPAP. Considering recent reports of *FOXO3a* gene expression being dysregulated in DTC [37], separation of FOXO3a transcriptional control from VD signaling should be focused in future studies as a potential oncogenic pathomechanism.

The impact of enzymatic SIRT1 activation or inhibition on thyroid cancer cell growth *in vitro* is poorly examined. Thus, while Shih et al. analyzed the effects of unspecific SIRT1 activator Resveratrol on proliferation and apoptosis of thyroid cancer cell lines [9], this is the first study to investigate the effect of SIRT1 inhibitor Ex-527. Whilst, Resveratrol induced apoptosis in thyroid cancer cells [9] we did not find effects of SIRT1 inhibition on cell proliferation of Thc and BCPAP cells.

## Conclusion

In conclusion, this is the first study that reports FOXO3a to be associated with autoimmune thyroid diseases. Thus, we could identify two SNP's that may constitute genetic risk factors for HT. Moreover, VD-SIRT1 interaction could be confirmed in DTC cells and immune cells, suggesting SIRT1 to be a crucial mediator of immune regulatory and anti-proliferative action and indicating SIRT-FOXO3a as downstream targets of VD effects. So far VD is not a component of DTC therapy or treatment of autoimmune thyroid diseases. A combination of VD with SIRT1 activators, such as resveratrol or SRT1720 [48], could be a promising approach for upcoming anti-proliferative therapies.

## Availability of data and material

All datasets for this study are included in the manuscript and the supplementary files.

## Author contributions

The studies subjects were recruited and the patients' blood samples were collected by FG, CV, CD, M-LH, WB, KH, and KB. NR performed the experimental setups. NR and MP-M analyzed and interpreted the patient data. NR was the major contributor in writing this manuscript, which was revised by KB and MP-M. All authors read and approved the final manuscript.

### Conflict of interest statement

The authors declare that the research was conducted in the absence of any commercial or financial relationships that could be construed as a potential conflict of interest. The reviewer SF and handling Editor declared their shared affiliation.

## Abbreviations

Ab: Antibody
CI: confidence interval
Co: control
DMSO: dimethylsulfoxide
DTC: differentiated thyroid cancer
FCS: fetal calf serum
GAPDH: Glycerinaldehyd-3-phosphat dehydrogenase
h: hours
HC: healthy controls
LD: linkage disequilibrium
OR: odds ratio
pc: corrected p-values
PHA: phytohemagglutinin
RT-PCR: real time polymerase chain reaction
SIRT1: Sirtuin1
SNP: single nucleotide polymorphism
Thc: T-helper cell
Tg: thyroglobulin
TPO: thyroperoxidase
UTR: untranslated
VD: vitamin D
VDR: vitamin D Receptor.
