# Supplementary material for: Vitamin D, FOXO3a, and Sirtuin1 in Hashimoto's Thyroiditis and Differentiated Thyroid Cancer
Source: Front Endocrinol (Lausanne). 2018 Sep 11;9:527. doi: 10.3389/fendo.2018.00527 (PMC6142903; doi:10.3389/fendo.2018.00527)
Supplement: Supplementary file 1 [file Table_1.docx]

**Supplemental table 1: Distribution of *FOXO3a* single nucleotide polymorphisms in differentiated thyroid carcinoma and healthy controls.**

| ***FOXO3a* rs4946936** | | | **Genotype/ n (frequency)** | | |  | |  |  | | **Allele/ n (frequency)** | | |  | |  | |  | |  |
| --- | --- | --- | --- | --- | --- | --- | --- | --- | --- | --- | --- | --- | --- | --- | --- | --- | --- | --- | --- | --- |
|  | **group n CC CT TT p p_c_ C T** OR [95% CI] C OR [95% CI] T **p  p_c_** | | | | | | | | | | | | | | | | | | | |
| HC 362 156(43.1%) 171(47.2%) 35(9.7%) 483(66.7%) 241(33.3%) | | | | | | | | | | | | | | | | | | | | |
|  | DTC 257 130(50.6%) 112(43.6%) 15(5.8%) 0.08 0.24 372(72.4%) 142(27.6%) 1.28[0.99-1.66] 0.78[0.6-1.01] **0.04 0.08** | | | | | | | | | | | | | | | | | | | |
|  | HC-♀ 208 89(42.8%) 99(47.6%) 20(9.6%) 277(66.6%) 139(33.4%) | | | | | | | | | | | | | | | | | | | |
|  | DTC-♀ 172 88(51.2%) 76(44.2%) 8(4.7%) 0.09 0.27 252(73.3%) 92(26.7%) 1.37[1.99-1.88] 0.73[0.53-1.00] 0.06 0.12 | | | | | | | | | | | | | | | | | | | |
| HC-♂ 154 67(43.5%) 72(46.8%) 15(9.7%) 206(66.9%) 102(33.1%) | | | | | | | | | | | | |  | |  | |  | |  | |
| DTC-♂ 85 42(49.4%) 36(41.4%) 7(8.2%) 0.67 2.01 120(70.6%) 50(29.4%) 1.19[0.79-1.78] 0.84[0.56-1.26] 0.47 0.94 | | | | | | | | | | | | | | | | | | | | |
| **FOXO3a rs4945816** | | | | **Genotype/ n (frequency)** |  | | | | | **Allele/ n (frequency)** | |  | | | | | | | | |
| **group n** | | | | **CC CT** | **TT p p_c_** | | | | | **C T** | | OR[95% CI] C OR[95% CI] T **p p_c_** | | | | | | | | |
| HC 353 31(8.8%) 153(43.3%) 169(47.9%) 215(30.5%) 491(69.5%) | | | | | | | | | | | | | | | | | | | | |
| DTC 246 13(5.3%) 106(43.1%) 127(51.6) 0.24 0.73 132(26.8%) 360(73.2%) 0.84[0.65-1.08] 1.19[0.92-1.54] 0.20 0.39 | | | | | | | | | | | | | | | | | | | | |
| HC-♀ 149 14(9.4%) 61 (40.9%) 71(49.7%) 89(29.9%) 209(70.1%) | | | | | | | | | | | | | | | | | | | | |
| DTC-♀ 171 8(4.7%) 71(41.5%) 92(53.8%) 0.21 0.63 87(25.4%) 255(74.6%) 0.78[0.55-1.1] 1.29[0.91-1.82] 0.19 0.37 | | | | | | | | | | | | | | | | | | | | |
| HC-♂ 204 17(8.3%) 92(45.1%) 95(46.6%) 126(30.9%) 282(69.1%) | | | | | | | | | | | | | | | | | | | | |
| DTC-♂ 83 7(8.4%) 35(42.2%) 41(49.4%) 0.90 2.69 49(29.5%) 117(70.5%) 1.07[0.72-1.58] 0.94[0.63-1.39] 0.82 1.64 | | | | | | | | | | | | | | | | | | | | |
| **FOXO3a rs9400239** | | | | **Genotype/ n (frequency)** | |  | | | | **Allele/ n (frequency)** | |  | | | | | | | | |
|  | **group n** | **CC CT** | | | | | **TT p p_c_** | | | **C T** | | OR[95% CI] C OR[95% CI] T **p p_c_** | | | | | | | | |
| HC 463 215(46.4%) 205(44.3%) 43(9.3%) 635(68.6%) 291(31.4%) | | | | | | | | | | | | | | | | | | | | |
| DTC 246 123(50.0%) 105(42.7%) 18(7.3%) 0.54 1.62 351(71.3%) 141(28.7%) 1.18[0.92-1.53] 0.85[0.92-1.53] 0.31 0.62 | | | | | | | | | | | | | | | | | | | | |
| HC-♀ 228 104(45.6%) 102(44.7%) 22(9.7%) 310(68.9%) 146(32.9%) | | | | | | | | | | | | | | | | | | | | |
| DTC-♀ 167 87(52.1%) 70(41.9%) 10(6.0%) 0.27 0.81 244(73.1%) 90(73.1%) 1.28[0.93-1.74] 0.78[0.57-1.07] 0.22 0.44 | | | | | | | | | | | | | | | | | | | | |
| HC-♂ 235 111(47.2%) 103(43.8%) 21(8.9%) 325(69.1%) 145(30.9%) | | | | | | | | | | | | | | | | | | | | |
| DTC-♂ 79 6(45.6%) 35(44.3%) 8(10.1%) 0.94 2.82 107(67.7%) 51(32.3%) 0.93[0.64-1.38] 1.07[0.73-1.57] 0.81 1.62 | | | | | | | | | | | | | | | | | | | | |

In total 257 patients with DTC and 463 HC were genotyped for SNP FOXO3a rs4946936, rs4945816 and rs9400239. None of the polymorphisms showed significant differences in genotype and allele distribution between patients and controls. However DTC tended to show higher frequency of allele “C” in comparison to HT.
